# A method to estimate statistical errors of properties derived from charge-density modelling

**DOI:** 10.1107/S2053273318004308

**Published:** 2018-05-03

**Authors:** Bertrand Fournier, Benoît Guillot, Claude Lecomte, Eduardo C. Escudero-Adán, Christian Jelsch

**Affiliations:** a Institut Galien Paris Sud, UMR CNRS 8612, Université Paris Sud, Faculté de Pharmacie, Université Paris-Saclay, 5 rue Jean-Baptiste Clément, Châtenay-Malabry, 92296, France; b Laboratoire Structures, Propriétés et Modélisation des Solides (SPMS) UMR CNRS 8580, Ecole CentraleSupélec, 3 rue Joliot-Curie, Gif-sur-Yvette Cedex, 91192, France; c CRM2, UMR CNRS 7036, Institut Jean Barriol, Université de Lorraine, Vandoeuvre les Nancy Cedex, France; d The Barcelona Institute of Science and Technology, Institute of Chemical Research of Catalonia (ICIQ), Avinguda Països Catalans 16, Tarragona, 43007, Spain

**Keywords:** Monte Carlo methods, electron density, uncertainty, topology, intermolecular interactions

## Abstract

Estimating uncertainties of property values derived from a charge-density model is not straightforward. A methodology, based on calculation of sample standard deviations (SSD) of properties using randomly deviating charge-density models, is proposed with the *MoPro* software. The parameter shifts applied in the deviating models are generated in order to respect the variance–covariance matrix issued from the least-squares refinement. This ‘SSD methodology’ procedure can be applied to estimate uncertainties of *any* property related to a charge-density model obtained by least-squares fitting. This includes topological properties such as critical point coordinates, electron density, Laplacian and ellipticity at critical points and charges integrated over atomic basins. Errors on electrostatic potentials and interaction energies are also available now through this procedure. The method is exemplified with the charge density of compound (*E*)-5-phenylpent-1-enylboronic acid, refined at 0.45 Å resolution. The procedure is implemented in the freely available *MoPro* program dedicated to charge-density refinement and modelling.

## Abstract 2

Errors on molecular properties including the topology of electron density and electrostatics are estimated from a sample of deviating models generated using the variance–covariance matrix issued at the end of the charge-density refinement.

## 1. Introduction   

Errors on electron-density-derived properties, such as topological characteristics or electrostatic potential, are generally poorly addressed in the relevant literature. To the best of our knowledge, no available computer software designed for charge-density analysis on the basis of multipolar modelling computes properly analytical standard deviations on electron-density-derived properties. For instance, in the *XD2006* program (Volkov *et al.*, 2006 ▸), there is a feature that allows one to compute estimated uncertainties of the electron density ρ(**r**), of the Laplacian ∇^2^ρ and of dipole moment values using the variance–covariance matrix, but it only accounts for the contributions of some of the parameters used in the Hansen & Coppens (1978 ▸) model, *i.e.* monopole and multipole populations. It implies that the propagation of errors due to the contributions of the atomic coordinates and of the contraction/expansion coefficients κ and κ′ is not taken into account. This could lead, consequently, to an overall underestimation of standard deviations on electron-density-derived properties.

Estimating uncertainties on properties derived from a charge-distribution model is yet essential to avoid any false or over-interpretation of these properties. When several experimental X-ray diffraction data sets collected during distinct and independent measurements are available for the same compound, it becomes possible to study the reproducibility of the refined charge-density model and to estimate uncertainties of derived properties through the determination of their sample standard deviations (SSDs). Such an approach was followed in a few studies, but often with questionable statistical significance given the sometimes very sparse sampling used [down to two models (Dittrich *et al.*, 2002 ▸; Grabowsky *et al.*, 2008 ▸), a larger sample (up to four data sets) but varying experimental temperatures or setups (Messerschmidt *et al.*, 2005 ▸; Förster *et al.*, 2006 ▸)].

Closely related but still different compounds (such as peptide bond properties in different amino acids) were also investigated (Flaig *et al.*, 1999 ▸). In an article dedicated to the transferability of atomic parameters in alanyl-*X*-alanine-type tripeptides, Grabowsky *et al.* (2008 ▸) computed the global average of the standard deviations (noted *experimental reproducibility indices* 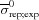) obtained in those studies, for various electron-density-derived properties of the QTAIM (quantum theory of atoms in molecules; Bader, 1990 ▸; Bader *et al.*, 1987 ▸) framework. For instance, they obtained, this way, average experimental errors 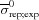 (ρ) = 0.07 e Å^−3^ and 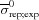 (∇^2^ρ) = 3.3 e Å^−5^ associated, respectively, with electron density and with Laplacian values at the bond critical points.

The most comprehensive and statistically sound reproducibility study on a wide range of electron-density-derived parameters was undertaken by Kamiński *et al.* (2014 ▸). They used 13 independently collected high-resolution X-ray diffraction data sets of α-oxalic acid dihydrate. From these data, obtained using similar experimental setups, they derived 13 oxalic acid charge-density models which were refined following identical strategies. This approach allowed them to analyse the normality of the error distribution in experimental data and in residual electron densities using the Shapiro–Wilk statistical test and, more importantly, to obtain very informative results in terms of dispersion of structural/charge-density model parameters and of charge-density-derived property values. They have shown, for instance, that among the multipole model parameters, the valence populations present large reproducibility deviations, reaching up to 40% of the corresponding atomic net charge. Conversely, multipole populations were characterized by moderate dispersions. Thus high reproducibility was achieved among the refined models. The multipole populations expected to be close to zero due to atom local symmetry were indeed statistically negligible. In the same way, concerning charge-density-derived properties, Kamiński *et al.* (2014 ▸) were able to evidence a significantly smaller dispersion of electron-density values on weak intermolecular (hydrogen bonds) critical points [10^−3^ < 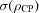 < 3 × 10^−2^ e Å^−3^] compared with covalent bonds [3 × 10^−2^ < 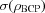 < 6 × 10^−2^ e Å^−3^] (CP = critical point, BCP = bond critical point) and, in any case, lower than the 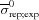 (ρ) value of 0.07 e Å^−3^ obtained by Grabowsky *et al.* (2008 ▸). The methodology proposed by Kamiński *et al.* (2014 ▸) provides standard deviations on any properties derived from the charge-density model, as well as possible rules of thumb for property uncertainties in any charge-density model of comparable quality. However, this approach is very resource- and time-consuming as it implies the collection of a statistically significant number of diffraction data sets at subatomic resolution. The uncertainties obtained may also not account totally for all systematic errors present in the data measurements.

Krause *et al.* (2017 ▸) recently presented a method based on *R*_free_ calculations. Sample standard deviations computed on the relevant models refined on subsets of the measured reflections (for example, 20 subsets of 95% reflections) can yield a rough estimate of the standard deviation on topological properties of the electron density. However, the *R*_free_ method has two drawbacks. Firstly, when strong reflections are omitted (put in the test set), the results of these refinements *versus* the remaining data are significantly influenced. This effect does not have much impact on the refinement of protein structures (which have poor *R* factors and a large number of reflections) but is crucial for the refinement of quantitative electron densities.

Secondly, the estimated uncertainty on a derived property obtained using this method depends on the number *N* of complementary *R*_free_ refinements performed. The discrepancy between the refined models decreases with *N*, as the number of free reflections omitted in the validation sets decreases proportionally to 1/*N*.

Here, we present a method allowing the estimation of uncertainties on properties derived from a charge-density model. This method consists of a statistical Monte Carlo random sampling procedure, based on the variance–covariance matrix obtained after the convergence of the least-squares refinement.

The least-squares method is widely used for the structural and charge-density refinement of crystal structures. The optimization procedure that uses the matrix of normal equations has a great power of convergence. The inversion of the full normal matrix also provides the variance–covariance matrix of the refined parameters and permits one to determine the precision of the refined structure model (Hamilton, 1964 ▸).

The current study addresses the uncertainty on properties related to the precision of measurements. The accuracy of properties which is related to systematic errors in measurements is however a different issue.

**Figure 1:**
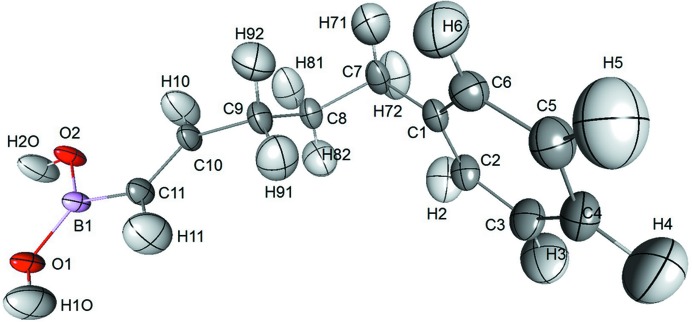
Structure of (*E*)-5-phenylpent-1-enylboronic acid.

In the present paper, the methodology for estimation of uncertainties is illustrated with the charge-density analysis of an organic compound: (*E*)-5-phenylpent-1-enylboronic acid (hereafter noted BOH2, Fig. 1 ▸). The unique electronic and physicochemical properties of boronic acid make this kind of compound very useful as a pharmaceutical agent. Boronic acids are strong Lewis acids. They can be used as enzyme inhibitors in Suzuki cross-coupling reactions, Diels–Alder reactions, carb­oxy­lic acid activation or selective reduction of aldehydes, among many other uses (Yang *et al.*, 2003 ▸). In recent years, boronic acids have also been reported as interesting building blocks in covalent organic frameworks (Côté *et al.*, 2007 ▸; Spitler & Dichtel, 2010 ▸; Ding *et al.*, 2011 ▸). To the best of our knowledge, this article is the first experimental charge-density study of a boronic acid compound.

## 2. Experiment   

### 2.1. Crystallization   

For the current experiment, crystals were grown by slow evaporation of an ethanol/water solution of the compound BOH2 in a few days at room temperature. A single, colourless crystal of dimensions 0.34 × 0.18 × 0.10 mm was selected for the diffraction experiment. The compound crystallized in the centrosymmetric space group *Pbca*. More data on the orthorhombic crystal of BOH2 are given in Table 1 ▸.

### 2.2. Data collection   

A single-crystal high-resolution and highly redundant X-ray data collection of the BOH2 compound was performed on a Rigaku MicroMax-HF rotating-anode diffractometer equipped with a Pilatus 200K hybrid pixel detector using Mo *K*α radiation (λ = 0.71073 Å). The crystal was mounted on a Kapton micromount. The data collection was carried out at 90 (1) K under a stream of nitrogen using the Oxford 700 Plus Cryosystems gas-flow apparatus.

The diffraction data were collected using ω scans of 0.5° intervals with the *CrystalClear-SM Expert 2.1b29* software (Rigaku, 2013 ▸) up to a resolution of 0.41 Å (sinθ/λ < 1.22 Å^−1^). The exposure times were 5 and 40 s per frame for low- and high-resolution data, respectively. Data reduction and absorption correction were performed using the *Crys­AlisPro 1.171.38.37f* package (Rigaku Oxford Diffraction, 2015 ▸); the internal *R*(*I*) factor was 3.06% for all reflections (Table 1 ▸).

### 2.3. Structure solution and refinement   

The structure of the BOH2 compound has already been determined (Gelbrich *et al.*, 2000 ▸). In our study, the structure of BOH2 was solved using the *SIR2014* software (Burla *et al.*, 2015 ▸). In particular all the H atoms were located in the difference Fourier map. An initial independent atom model (IAM) refinement was undertaken using the *SHELXL2014* software (Sheldrick, 2015 ▸).

### 2.4. Multipolar refinement   

The charge-density model was refined against diffraction intensities using the program *MoPro* (Guillot *et al.*, 2001 ▸; Jelsch *et al.*, 2005 ▸). The program is based on the multipolar scattering factor formalism of Hansen & Coppens (1978 ▸) and allows the definition of restraints on stereochemistry, thermal motion and charge-density parameters. Data resolution was truncated at 0.45 Å as the very high resolution reflections showed decreasing values of 〈*F*_o_^2^〉/〈*F*_c_^2^〉 well below unity, as verified with the *XDRK* software (Zhurov *et al.*, 2008 ▸). For the same reason, an *I*/σ_*I*_ > 0.35 cutoff was applied. The evolution of 〈*F*_o_^2^〉/〈*F*_c_^2^〉 as a function of reciprocal resolution *s* is shown in the supporting information.

The multipole expansion was done at the octupolar level for B, C and O atoms and the dipole level for H atoms. The core and valence spherical scattering factors were calculated using the wavefunctions for isolated atoms from Su & Coppens (1998 ▸) and the anomalous dispersion coefficients were taken from Kissel *et al.* (1995 ▸).

The *MoPro* program has numerous functionalities with respect to constraints, restraints and similarity applying to the stereochemistry and charge density. For the H atoms, the values of anisotropic *U_ij_* parameters were fixed to those obtained from the SHADE3 server (Madsen & Hoser, 2014 ▸). The H—*X* distances of H atoms were restrained to the values obtained from neutron diffraction studies (Allen & Bruno, 2010 ▸) with a restraint sigma σ_rest_ of 0.01 Å. Distance *X*—H similarity restraints were also applied to chemically equivalent groups (σ_rest_ = 0.01 Å).

The charge-density model was subsequently refined against diffraction intensities. The electron-density maps, local topological properties and intermolecular electrostatic energies were computed using the *VMoPro* module of the *MoPro* suite (Guillot *et al.*, 2001 ▸; Jelsch *et al.*, 2005 ▸), while the molecular view with thermal ellipsoids and the isosurface representations were produced with *MoProViewer* (Guillot *et al.*, 2014 ▸).

Automatic restraints of chemical equivalence and local symmetry (Domagała & Jelsch, 2008 ▸) were applied to the electron-density parameters such as contraction/expansion κ and κ′, valence and multipole populations *P*_val_ and *P*_*lm*_. The optimal weight σ_opt_ of the restraints applying to the charge-density parameters (atom equivalence and local symmetry) was set to 0.2, as determined by minimizing the global *R*_free_ factor (Brünger, 1992 ▸; Zarychta *et al.*, 2011 ▸). The parameters κ and κ′ of H atoms were restrained to be similar (σ_rest_ = 0.02).

The molecular parameters including scale factor, *xyz*, 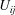, *P*_val_, *P*_*lm*_, κ and κ′ were refined together with the block diagonal option and finally using the full normal matrix until convergence, yielding *wR* ^2^(*I*) = 3.6%. The crystallographic details of the refinement are given in Table 1 ▸.

The topological charges were integrated on atomic basins using the program *BADER* (Tang *et al.*, 2009 ▸). A parallelepiped embedding the BOH2 molecule extracted from the crystal lattice was defined with a margin of 3 Å around the atomic nuclei. For each deviating model, the total electron density of the molecule inside this parallelepiped was computed using the program *VMoPro*, with a grid step of 0.05 Å along each direction and then saved as a Gaussian cube file. Then, the program *BADER* was used for atomic basin definition and charge integration. The sum of the integrated electron charges was smaller than the total number of electrons in the molecule with an average lack of 0.47 e (SSD = 0.0028 e) for a total number of 102 electrons. The unattributed electron charge was evenly redistributed on the 29 integrated atomic basin charges.

## 3. Methodology   

### 3.1. Least-squares refinement and uncertainties   

The least-squares refinement is implemented in *MoPro* (Guillot *et al.*, 2001 ▸; Jelsch *et al.*, 2005 ▸), software dedicated to charge-density refinement. A multipolar charge-density model defined according to the formalism of Hansen & Coppens (1978 ▸) can be refined for crystal structures when ultra high resolution X-ray diffraction data have been measured. For macromolecular structures, the transferability principle can be used to define a multipolar electron-density model. When the refinement is performed against the reflection intensities, the minimized function *E* is defined as
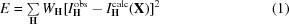
where 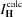 and 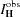 are the calculated and observed reflection intensities, respectively, and 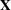 is the vector of the model parameters being considered in the corresponding refinement stage. The factor *W* **_H_** represents a weight for each reflection **H**. This weight can be taken as the squared inverse estimated error of the measured intensity.

The structure-factor amplitude is obtained by summation over all atoms *a* in the asymmetric unit and all symmetry operators *s* of the space group, as follows: 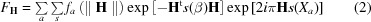
where, for each atom *a*, *f*_*a*_ is the atom scattering factor, β is the dimensionless thermal tensor and *X*_*a*_ is the atom coordinates.

The least-squares refinement is performed iteratively. At each refinement cycle of *n* parameters in the model and after linearization of the calculated reflection intensities around the current vector 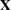, the minimization of *E* is performed by solving the matrix system of normal equations: 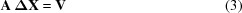
where 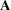 is the 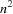 symmetric normal matrix, 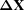 is the unknown shift vector to apply to the *n* variables refined. **V** is a vector of dimension *n* with elements like 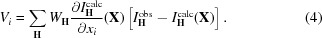
The normal matrix element 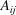 concerning the refined parameters *x*_*i*_ and *x*_*j*_ is obtained from the summation of the products of the intensity (or structure-factor) derivatives over the reflections **H**: 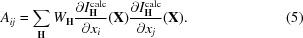
The normal matrix elements [equation (5)], through the calculated intensities, incorporate implicitly the contribution of symmetry-related atoms in the unit cell as can be seen in equation (2). At the refinement convergence, the variance–covariance matrix of the model parameters is obtained from **B**, the inverse of matrix **A** (Hamilton, 1964 ▸). The *i*th diagonal term of the matrix **B** provides an estimated standard deviation (e.s.d.), noted σ(*x_i_*), of the parameter *x_i_*. If the weighting scheme *W* **_H_** used in the least-square function is not properly scaled, all e.s.d.’s have to be multiplied by the goodness of fit (GOF ≠ 1): 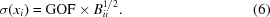
The correlation coefficient 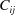 between the parameters 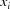 and 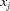 in the refinement is obtained by the equation 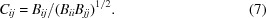



### 3.2. Generation of randomly deviating charge-density models   

The procedure is started from the converged charge-density model at **X** ^min^. The values of the parameter vector **X** are assumed to be distributed according to a multidimensional Gaussian probability density function with mean 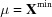 and variance–covariance matrix 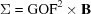.

If there were no correlations between parameters, the matrices **A** and **B** would be diagonal and the shifts *dx*_*i*_ to apply to each parameter to obtain a deviating model would be the e.s.d.’s 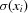 multiplied by a random number: 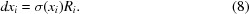
In other words, 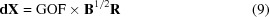
where **R** is a vector of random and independent real numbers normally distributed with a zero average and a unitary variance.

In real situations, the variance–covariance matrix **B** is symmetric positive-definite but not diagonal as parameters show some correlations. The deviating parameter vector **X** values are generated using the following practical procedure.

Since the normal matrix **A** is symmetric definite-positive, the matrix **A** is orthogonally diagonalized at the 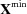 value where *E* is minimal leading to the expression 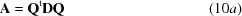
where **Q** is an orthogonal matrix and **D** is diagonal which contains the strictly positive eigenvalues of **A**.

Therefore, its inverse matrix **B** can be written as 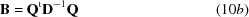
and the matrix **S**, a square root matrix of **B**, can be obtained as 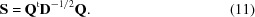
The deviating parameter vector **X** is obtained by applying 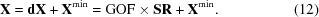
Each element of the vector **dX** is a linear combination of the **R** elements and thus the vector **X** follows a multivariate Gaussian distribution. The mean vector 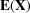 of this distribution is equal to 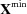: 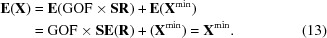
By propagation of uncertainties, the variance–covariance matrix of this multivariate Gaussian distribution is defined, using expression (9), as 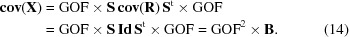
The events of the normal distribution of the vector **R** are generated using a random Gaussian number generator with zero mean and unitary sigma. The software *MoPro* generates random Gaussian numbers using the ‘ratio of uniform deviates’ method introduced by Kinderman & Monahan (1977 ▸) and augmented with quadratic bounding curves by Leva (1992 ▸). To avoid rare events which would lead to meaningless deviating charge-density models, the algorithm is modified to generate random numbers following a truncated Gaussian function. This modification consists of reducing the infinity support of the Gaussian probability density function to a [−4; 4] interval and of normalizing the resulting function in order to obtain a unitary variance.

Following the Monte Carlo procedure described in this section, several deviating charge-density models are generated using equation (12). The studied properties are computed on all these models and the SSDs are deduced from the sample values. The method is applied in the current study to the BOH2 molecule.

The number of deviating models required depends on the expected precision of SSDs. For any property *P*, assuming it follows a normal distribution with 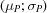, if a sample of *N* events, 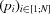, is taken from its distribution, the SSD, estimator of 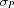, can be defined as 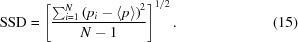
The quantity 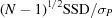 follows a χ probability distribution with 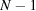 degrees of freedom (for more details, see Appendix *A* [App appa]). This implies that the expected relative standard deviation of the estimator SSD can be approximated as follows: 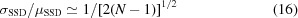
where 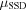 is the expected uncertainty value and 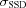 the standard deviation of the estimator SSD. We can select a number of events large enough to have an expected relative standard deviation value [equation (16)] smaller than a limit value 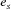 (see Fig. S1 in the supporting information). This information is relevant to estimate the number of deviating models necessary for a proper estimation of model property uncertainties. Expression (16) is however only strictly valid for a sample of random values from a normally distributed population. It is used in our study to estimate the uncertainty of the SSD for derived properties, assuming that their distributions are normal. For example, using *N* = 20 deviating models, for any derived property SSD, a relative standard deviation of 16% is expected. This precision is enough to estimate standard deviations of the considered properties with one significant digit.

The method is tested on the charge-density model of the BOH2 molecule, by generating a series of 20 randomly deviating models from which various derived properties are calculated, along with their SSDs.

For some examples of derived properties, a larger sample of 500 models has been used to obtain population histograms and to check the nature of population distributions. These histograms (Laplacian and ellipticity at the bond critical point, electrostatic energy) are provided in Fig. S2. It appears almost all properties have unimodal and Gaussian-like population distributions. The histogram of *wR* ^2^(*I*) factors is also shown; the value for the refined model is 3.616%, while for the perturbed structures the *R* values are always higher and the average *wR* ^2^(*I*) is 3.694% with a SSD of 0.007.

## 4. Results   

### 4.1. Geometry properties: distances and angles   

The good accordance between SSD and e.s.d. values has been verified for the parameters used to describe the structure and charge density. For example, Fig. S3 shows the agreement between e.s.d.’s issued from the least-square normal matrix inversion using equation (6) and the SSD values obtained from 20 deviating structures for the atomic fractional coordinates.

The SSD has been calculated for the bond distances and angles. For comparison, e.s.d.’s have also been retrieved from the error propagation method implemented in the *MoPro* software, and the relative differences between the SSD and e.s.d., |SSD − e.s.d.|/e.s.d., are calculated to check the reliability of the error propagation method.

**Figure 2:**
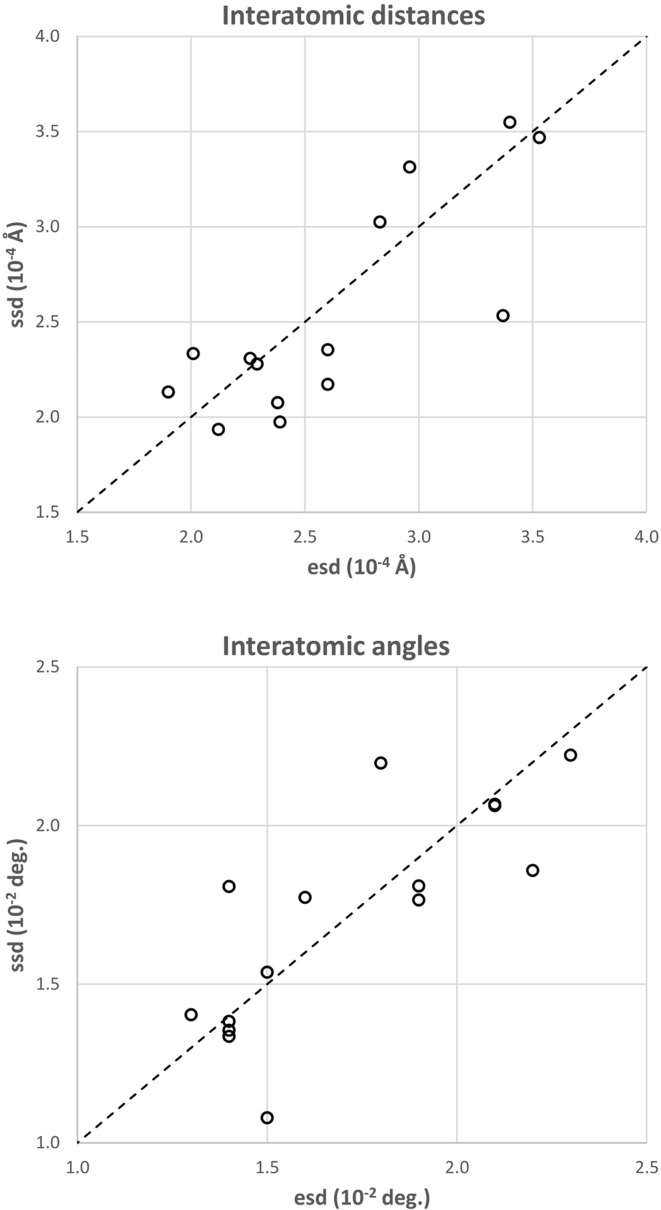
Scatter plot of the sample standard deviations (SSDs) *versus* the estimated standard deviations (e.s.d.) computed with *MoPro* for interatomic distances between non-H atoms, and angles between three non-H atoms. The first bisector is plotted as the black dashed line.

In the plots SSD *versus* e.s.d. for the interatomic distances and angles between non-H atoms (Fig. 2 ▸), the points are distributed along the *y* = *x* line. Moreover, the maximal value of 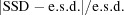 is 25% for interatomic distances and 30% for interatomic angles, which implies a good agreement between SSD and e.s.d. if only one significant digit is expected.

### 4.2. Electron density   

**Figure 3:**
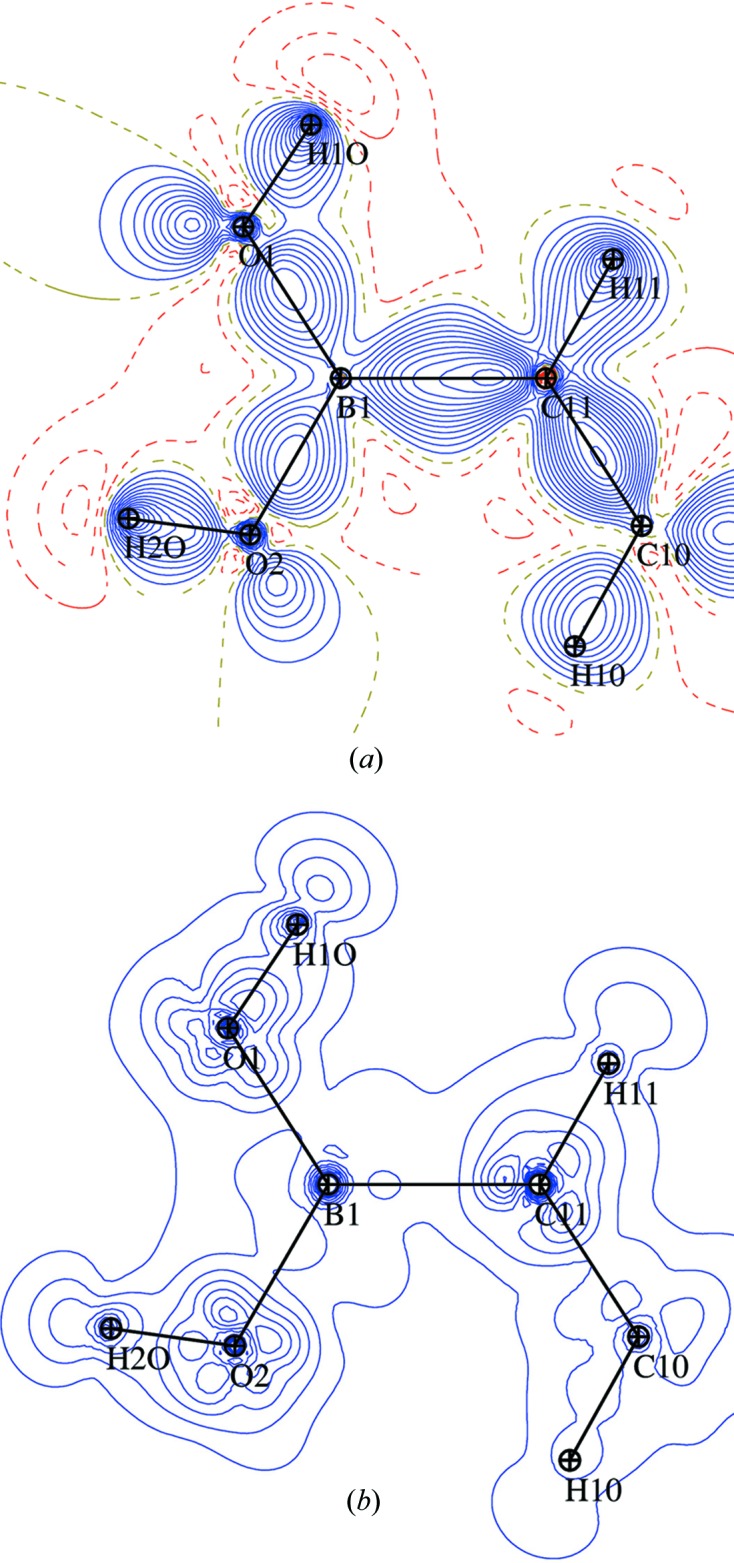
Static deformation electron density in the (C11, B1, O2) plane. Atoms C9, C10 and O1 are also in the plane. (*a*) Deformation with contours of ± 0.05 e Å^−3^. Blue solid line, positive; red dotted lines, negative. (*b*) Sample standard deviation, SSD, of the deformation density deduced from 20 models with contours of ± 0.005 e Å^−3^.

The statistical procedure used to estimate standard deviations can be extended to any molecular property, including the static electron density. The static deformation electron density in the (C11, B1, O2) plane is considered as an example in Fig. 3 ▸(*a*). The SSD map (Fig. 3 ▸ *b*) in the (C11, B1, O2) plane shows significant features near atomic nuclei, which is expected as the electron density takes large values and varies drastically in their vicinity with the nuclei coordinate shifts. These features around nuclei are anisotropic, which can be related to the positive and negative multipolar deformation density in the map (Fig. 3 ▸ *a*) due to the formation of covalent bonds or the electron lone pairs in the O-atom case.

The SSD(ρ) level is found to be below 0.015 e Å^−3^ on the covalent bonds between non-H atoms; for bonds involving H atoms SSD(ρ) is, in comparison, slightly higher, but still below 0.020 e Å^−3^.

### 4.3. Topology of covalent bonds   

For each deviating model, covalent bond topological analysis is performed using the software *VMoPro* and the results processed by statistical analysis for SSD estimation. For each covalent bond, the distances between bonded atomic nuclei and the corresponding BCP position are reported, with the topological properties, in Table 2 ▸.

The intramolecular bonds involving the B atom have the largest uncertainties on the Laplacian values. The B—O bonds in particular show positive ∇^2^ρ_CP_ Laplacian values and the largest uncertainties among covalent bonds between non-H atoms, as boron is a very light element with respect to oxygen. The B—O bonds also have the most accurate distances *X*—CP and *Y*—CP with uncertainties below 10^−3^ Å (Table 2 ▸). Among the *X*—H bonds, the ones with O atoms have the largest (in magnitude) Laplacian values and SSDs; the relative uncertainties are however similar, around 2.4% for all *X*—H bonds (Table 2 ▸).

**Figure 4:**
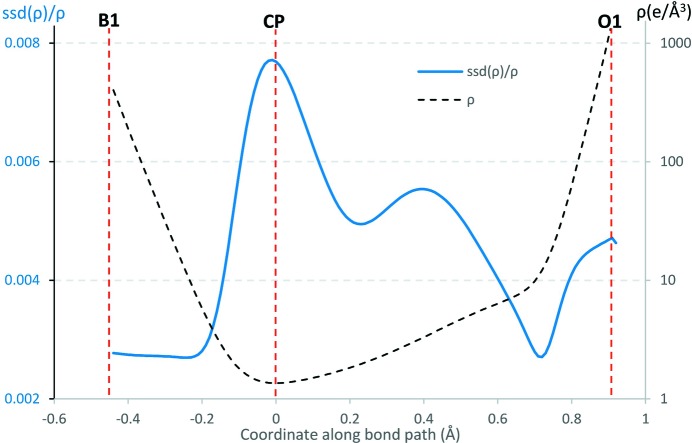
Plots of the electron density ρ and its relative SSD along the B1—O1 bond path. The ρ plot is shown in logarithmic scale.

Uncertainties of electron densities ρ and Laplacian values ∇^2^ρ at the CPs show, in the case of *X*—*Y* bonds (hereafter, *X* and *Y* stand for non-H atoms), average values around 0.010 e Å^−3^ and 0.42 e Å^−5^ while their maxima reach, respectively, 0.014 e Å^−3^ and 0.93 e Å^−5^. In the case of *X*—H bonds, the average uncertainties of ρ and ∇^2^ρ are quite comparable with the previous ones, with, respectively, 0.014 e Å^−3^ and 0.42 e Å^−5^ and maximal values of 0.031 e Å^−3^ and 0.98 e Å^−5^. It must be noted that, in both cases, uncertainties are dramatically below the root mean square discrepancies reported by Grabowsky *et al.* (2008 ▸) in a study of the charge densities of peptides. The SSD(ρ_CP_) values are in accordance with those found in the SSD map of the static deformation density (Fig. 3 ▸ *b*). The ρ electron density and its relative SSD are shown in Fig. 4 ▸ along the B1—O1 bond path and the SSD(ρ) error is two orders of magnitude smaller than ρ. The errors on ρ on the B1—O1 bond are comparatively lower than those on the C—O bond of oxalic acid exemplified in the Kamiński *et al.* (2014 ▸) study. The mean error over density 〈SSD(ρ)/ρ〉 is 1% while for the Laplacian 〈SSD(∇^2^ρ)/∇^2^ρ〉 reaches 3%.

The SSD values of the (λ_1_, λ_2_, λ_3_) Hessian matrix ∂^2^ρ/∂*x* *_i_*∂*x*_*j*_ eigenvalues at the bond CPs are shown in Table S1. Examples of population histograms for the Laplacian and ellipticity at the CP of the bond B1—O1 are shown in Figs. S2(*a*), S2(*b*). It is relevant to note that the ellipticity at an electron-density CP, which is, by definition, positive, can have a drastically asymmetric statistical density distribution when its reference value derived from the converged model is small relative to its SSD (Fig. S2*b*).

**Figure 5:**
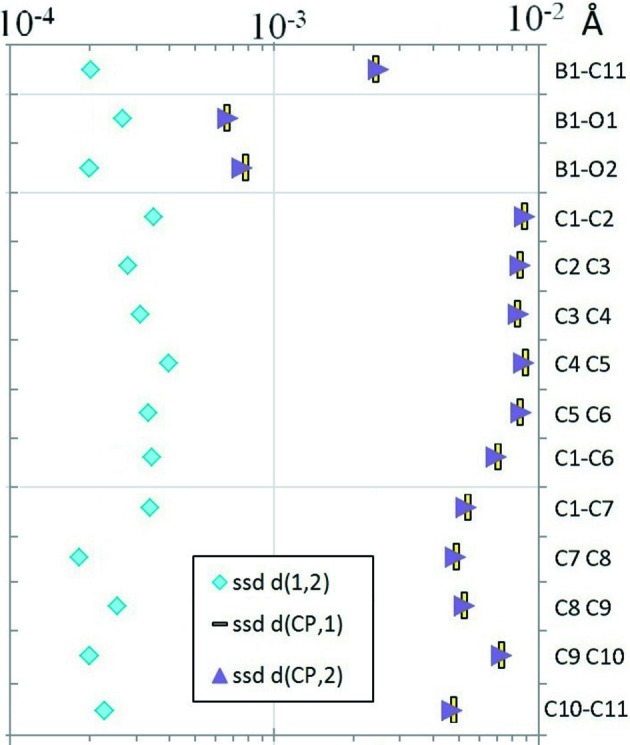
Plot of SSD values of *X*⋯CP and *Y*⋯CP distances for all *X*—*Y* covalent bonds between non-H atoms. The SSDs of the *X*—*Y* bond distances are also shown.

The plot of SSD values of distances *X*⋯CP *versus* *Y*⋯CP for the *X*—*Y* covalent bonds (non-H atoms) is illustrated in Fig. 5 ▸. A remarkable equality between uncertainties in the distances *X*⋯CP and *Y*⋯CP can be observed, the SSDs being generally in the 2 × 10^−4^–4 × 10^−4^ Å range. This result can be simply explained by the high accuracy of heavy-atom nucleus positions relative to BCP positions, making the uncertainty of the CP position the predominant cause of error. This is confirmed by the lower order of magnitude of the *X*—*Y* distance uncertainties compared with the ones on distances *X*⋯CP and *Y*⋯CP (Fig. 5 ▸, Table 2 ▸).

The *X*⋯CP and H⋯CP distance SSDs involving *X*—H bonds are higher, mostly in the 4 × 10^−4^–8 × 10^−4^ Å range (Fig. S4). The observed SSD values of *X*⋯CP and H⋯CP distances are, in this case, more dissimilar, but of the same order of magnitude as the *d*(*X*, H) SSD. It has to be recalled here that H-atom positions were restrained during the model refinement (§2.4); therefore the *d*(*X*⋯H) values and their uncertainties obtained depend partly on the distance restraints used.

**Figure 6:**
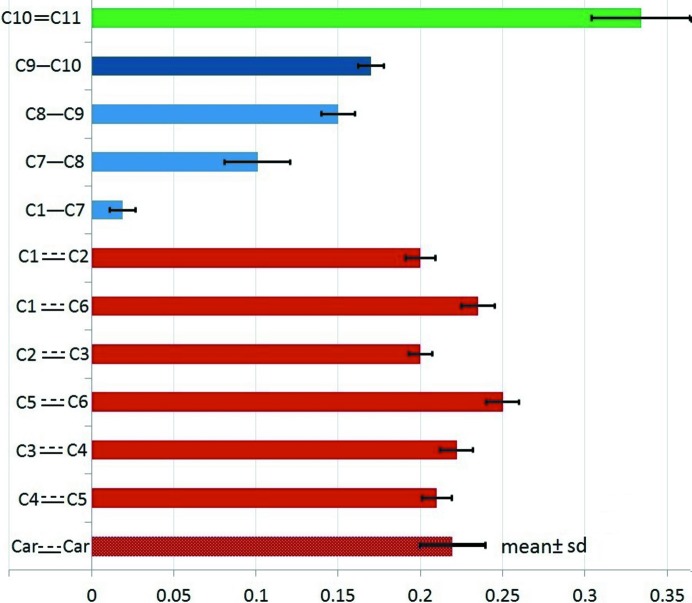
Histogram showing the ellipticity of the C—C bonds. Error bars correspond to the SSD values. The average and root mean square deviation (r.m.s.d.) for the six C—C bonds in the aromatic cycle are also shown. Bonds are distinguished by type: aromatic in red, double in green and single in blue.

The knowledge of uncertainties is crucial to assess the pertinence of discussions on the property values. For instance, the histogram of ∊ ellipticities with SSDs on the C—C bond CPs allows one to compare the values visually (Fig. 6 ▸). With respect to SSD values, the formally double bond C10=C11 clearly has a higher ellipticity than all other bonds. Among the four formally single bonds, the differences between ∊ values are generally significant as the standard deviation between values (0.067) is 5.7 times larger than the average SSD uncertainty (0.012). The discrepancies among the aromatic bonds are less meaningful with a standard deviation between values of 0.020, which is only two times larger than the average SSD uncertainty (0.011).

### 4.4. Topology of intermolecular interactions   

Intermolecular interactions play a key role in crystal engineering which is an important field in chemical crystallography; therefore estimation of errors on their properties is extremely timely. In the BOH2 crystal packing, 17 unique interatomic contacts shorter than 3 Å were identified between the reference molecule and its environment, involving eight distinct neighbour molecules (Table 3 ▸). The intermolecular (3,−1) CP search has been done using the software *VMoPro* on the 20 deviating models. All the O⋯H hydrogen bonds show non-ambiguous bond paths between the two atoms. Two of the intermolecular contact CPs have unstable bond paths, in the sense that they lead to different linked atoms within the deviating models (Table 3 ▸). The first non-stable bond path involves the phenyl H4 atom of the molecule (−*x* + 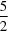, *y* − ½, *z*) which is connected to the phenyl C atoms of the reference molecule, C1 in 13 deviating models and C6 in the seven others. The second ambiguous bond path involves another weak phenyl⋯phenyl interaction between the H3 atom of the reference molecule and either C4 (three in 20 cases) or H4 of the molecule (*x* − ½, *y*, −*z* + ½) (17 in 20 cases). The C⋯H contacts can be considered as very weak hydrogen bonds [respectively, ρ = 0.0364 (8) and ρ = 0.0432 (9) e Å^−3^] with the phenyl moiety as acceptor. Moreover, two reported van der Waals contacts between H atoms, at *d*(H⋯H) > 2.7 Å, yield a CP and bond path detected only in some of the deviating models and are reported in italics in Table 3 ▸. Globally, the bond paths and CPs are found to be stable in the models perturbed at standard deviation in all the strongest interactions and most of the weaker ones.

For the properties at the intermolecular bond CPs which are systematically detected in all models, the uncertainties of ρ_CP_ and Laplacian ∇^2^ρ_CP_ values are of the same magnitude as those shown by Kamiński *et al.* (2014 ▸) and do not exceed 6 and 4% in relative values, respectively. Similar uncertainty could also be observed in the intermolecular area from the static deformation density SSD map (Fig. 3 ▸ *b*), where SSD values tend to be lower than the 0.005 e Å^−3^ contour level outside of the molecule.

The mean error over density at the intermolecular CPs 〈SSD(ρ)/ρ〉 is 3%. For the Laplacian, the mean 〈SSD(∇^2^ρ)/|∇^2^ρ|〉 is 3.7% on the two hydrogen bonds while it reaches only 1.3% on the weaker interactions. The relative errors are similar for ρ_CP_ on the covalent bonds and non-bonded interactions. Conversely, Laplacian values generally have a lower relative error on weak interactions compared with strong hydrogen bonds or covalent bonds.

The SSD of *G*_CP_ and *V*_CP_, the kinetic and potential energy density (Espinosa *et al.*, 1998 ▸), respectively, derived from ρ_CP_ and ∇^2^ρ_CP_, can also be computed. The dissociation energy *E*_HB_ = −*V*_CP_ of the two O⋯H—O hydrogen bonds present in the BOH2 crystal packing was estimated. For O1⋯H2*O*, *E*_HB_ = 37.9 (9) and for O2⋯H1*O*, *E*_HB_ = 41 (2) kJ mol^−1^; the relative errors are therefore 2.3 and 5.6%, respectively.

### 4.5. Atomic charges   

**Figure 7:**
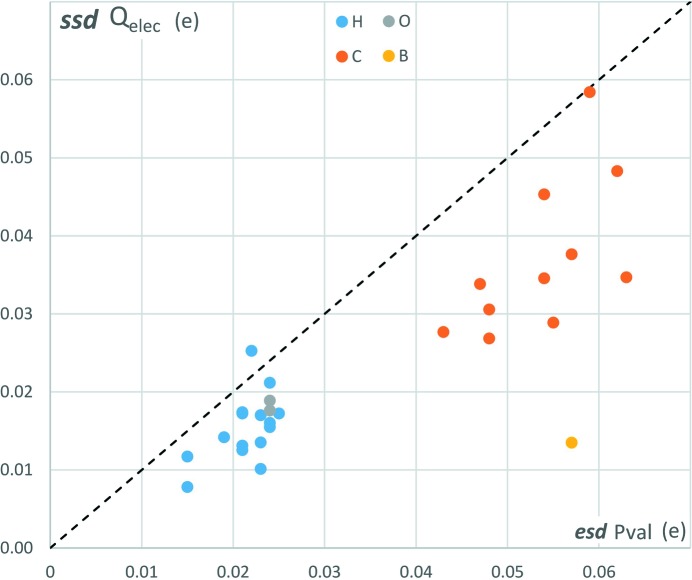
Sample standard deviation (SSD) of atomic basin electronic charge *Q*_elec_ plotted *versus* the estimated standard deviation (e.s.d.) of atomic valence population *P*_val_.

The atoms in molecules (AIM) topological analysis is extended to the integrated topological properties. The series of topological analysis results are used to estimate the uncertainties of atomic basin charges (Table 4 ▸). The integrated charge SSDs are found to be higher for C atoms (0.03 to 0.06 e) than for H, B and O atoms in the current structure (below 0.02 e in general). The average SSD value over all atomic charges is 0.024 e and the maximal SSD is obtained for the C5 atom of the phenyl moiety with 0.058 e. Such values are smaller but of the same order of magnitude as the typical uncertainties of atomic valence populations obtained from the variance–covariance matrix at the end of the multipolar refinement (Table 4 ▸). The SSDs of atomic basin electronic charges *Q*_topo_ are plotted against the e.s.d. of atomic valence populations *P*_val_ (Fig. 7 ▸). The valence population e.s.d. and SSD values are in good agreement (Table 4 ▸, Fig. S9). For most of the atoms, the valence population e.s.d. and SSD values are larger than the SSD of the corresponding integrated atomic basin charge. However, as SSD values of topological charges are consistently around a few tenths of an electron while their values can vary by several orders of magnitude (between 3 × 10^−3^ e for H72 and 2.4 e for the B atom), the corresponding relative uncertainties of *Q*_topo_ atomic charges can reach high values, especially for atoms bearing low integrated charges. For some H atoms, the uncertainty is larger than their weak charge (H71, H72, H82, H91) (Table 4 ▸). For the O atoms which bear a negative *Q*_topo_ charge of about −1.3 e, the relative error 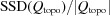 reaches on the other hand only 1.4%. The strongly positive atomic charge *Q*_topo_ of the B atom bonded with these two O atoms leads to a low relative error of 0.6%.

The two definitions of charges *Q*_topo_ and *P*_val_ derived are generally in good agreement, except for the B and O atoms which show much larger *Q*_topo_ charges. When the charge integration is carried out on the pro-molecule with spherical neutral atoms (IAM), the B atom turns out to have *Q*_topo_ = +1.60 e charge, while for the O1 atom *Q*_topo_ is −1.04 e, values which are far from the zero charge of a neutral atom. Therefore, the raw topological charges are not always to be compared with the *P*_val_ derived charges when atoms with very dissimilar atomic numbers, such as B and O, form a covalent bond. Non-zero *Q*_topo_ charges were recently reported by Stachowicz *et al.* (2017 ▸) for a CaF_2_ crystal when using the IAM model.

### 4.6. Electrostatic potential   

**Figure 8:**
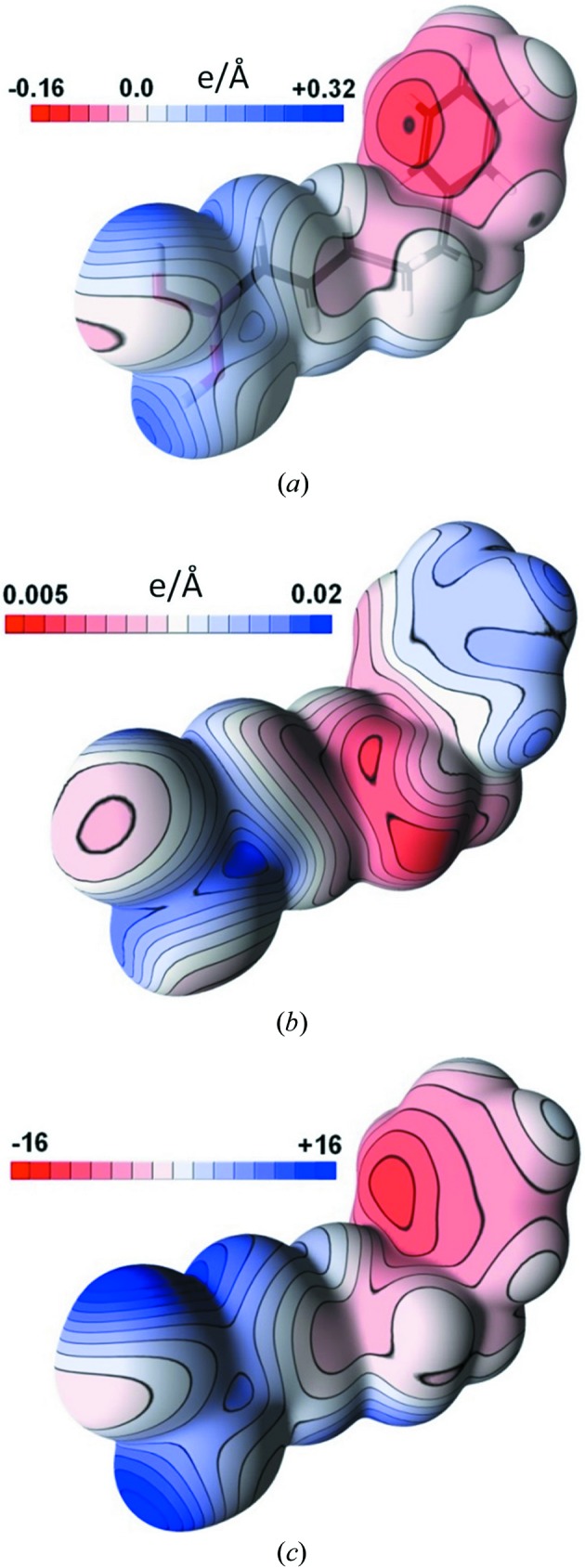
Electrostatic properties mapped on the 0.001 a.u. electron-density surface of the BOH2 compound. (*a*) Electrostatic potential φ, (*b*) SSD(φ) and (*c*) electrostatic potential divided by the SSD value [φ/SSD(φ)].

The 0.001 a.u. (a.u. = atomic units) electron-density iso­surface of the isolated molecule was chosen to map the molecular electrostatic potential Φ and its sample standard deviation 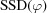 (Fig. 8 ▸ *a*). On this surface, the SSD of the electrostatic potential on the molecular surface lies between 5 × 10^−3^ and 2 × 10^−2^ e Å^−1^ and the average ‘signal over uncertainty’ ratio 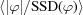 reaches 4.8. As depicted in Fig. 8 ▸ and Fig. S5, there is no clear correlation between the electrostatic potential SSD on the isosurface and its absolute value on the electron-density isosurface (correlation coefficient = 17%). Regions of highest 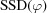 can be seen nearby the H2, H3 and H4 phenyl ring H atoms and close to the B atom (blue patches on Fig. 8 ▸ *b*). These locally large 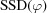 values can be explained by the fact that these H atoms present the largest thermal displacement parameters (2.9 < *B*_eq_ < 3.2 Å^2^) in the BOH2 compound, leading to larger uncertainties on their positions. Similarly, the high 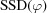 values observed in the vicinity of the B atom can be explained by an e.s.d. on its valence population that is twice as large as those of their neighbour O atoms (Table 4 ▸), locally increasing the SSD of the molecular electrostatic potential. Molecular surface points which are mostly under the electrostatic influence of these atoms show consequently particularly large 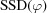 values. Nearly 90% of the considered surface points present 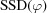 values lying between 0.008 and 0.016 e Å^−1^, distributed around the 0.012 e Å^−1^ average value and spanning the whole electrostatic potential values range (−0.16 to +0.32 e Å^−1^). The three-dimensional distribution of 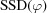 values is presented in Fig. S6 by the mean of three superimposed 0.04, 0.02 and 0.01 e Å^−1^ isosurfaces. As expected, the 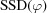 increases strongly in close vicinity to the atomic nuclei, where electrostatic potential variations become large due to the perturbed nuclei positions and valence populations in the 20 considered models contributing to the statistics. The volume of space located between the 0.01 and 0.02 e Å^−1^ isosurfaces of 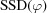 encompasses typical intermolecular interaction distances, *i.e.* regions where electrostatic potential is usually interpreted. The 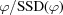 ratio is useful to estimate the electrostatic potential statistical significance on various regions of the electron-density surface (Kamiński *et al.*, 2014 ▸). This property, mapped on the electron-density surface, is represented in Fig. 8 ▸(*c*). The electrostatic potential is therefore statistically very significant in regions of strong values, with 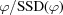 reaching 16 in our case. Conversely, 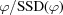 becomes lower than unity when the electrostatic potential falls below ∼0.02 e Å^−1^, in absolute value, which can be interpreted as a broadening of the zero potential contour regions on the molecular surface, as represented in white in Fig. 8 ▸(*c*) using a significance criterion of 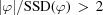. Regions located either side of this low-potential stripe can then be considered as either electropositive or electronegative with a high degree of confidence.

### 4.7. Electrostatic energy   

Eight unique dimers of molecules in contact have been identified in the crystal packing. Considering each dimer, the intermolecular electrostatic energy is computed for the 20 perturbed models using the EP/MM method (Volkov *et al.*, 2004 ▸) as implemented in the software *VMoPro*, and the SSD is calculated as the uncertainty estimator (Table 5 ▸).

Absolute errors of intermolecular electrostatic interaction energies in dimers of BOH2 molecules appear consistently of a few kJ mol^−1^. Therefore the SSD relative error is as low as 7% for the largest value *E*_elec_ = −62.2 kJ mol^−1^ but for the weakest interactions the SSD is larger than *E*_elec_ itself. Such large relative errors confirm clearly that weak electrostatic interaction energies of a few kJ mol^−1^ cannot be interpreted as either stabilizing or destabilizing. This is perfectly in line with the chemical accuracy in computational chemistry, generally considered to be around 5 kJ mol^−1^ (or 1 kcal mol^−1^) (Perdew *et al.*, 1999 ▸). For the energy summed over all dimers, the error reaches 19%. As the energy value results from an integration product between the electron density and the electrostatic potential, the relative errors of the two factors accumulate to yield a larger relative error.

Examples of Gaussian-like population histograms for electro­static interaction energies are shown in the supporting information for symmetry operations (*x* − 1, *y*, *z*) and (−*x*, −*y* + 2, −*z*) (Fig. S2).

### 4.8. Parameters to take into account   

In the method proposed, the generation of a series of deviating models is done by the calculation of the square root matrix **S** [equation (11)] which is obtained after diagonalization of the full normal matrix **A** whatever the derived property of interest. In practice, the procedure bears some similarity to a refinement step [equation (3)], but the inverted normal matrix **B** is replaced by its square root **S** [equations (11) and (12)] and the vector **V** is replaced by random numbers **R**.

The SSDs of the dimers’ electrostatic energy obtained from 20 deviating models generated starting with the reduced least-squares normal matrix obtained excluding the contributions of the *U*_*ij*_ thermal displacement parameters are also shown in Table 5 ▸. Nearly all these SSDs are smaller compared with the standard procedure where the full normal matrix is used. The SSD of the total *E*_elec_ value is significantly reduced from 14.8 to 8.9 kJ mol^−1^ when thermal displacement parameters are excluded from the normal matrix. Although the *U*_*ij*_ parameters are not directly involved in the equation describing the static electron density and the electrostatic potential, they do have an impact on the magnitude of SSD values.

This is due to the properties of the inversion of the symmetric positive-definite matrix. It is demonstrated in Appendix *B* [App appb] that, when more parameters are refined, the diagonal elements of the inverted normal matrix **B** = **A** ^−1^ take larger values. Consequently, when the number of refined parameters is increased, the e.s.d.’s of parameters become larger and the SSDs of derived properties also tend to increase. This is especially the case when there are significant correlations between parameters. Obtaining very high resolution in the diffraction data set tends actually to globally diminish the correlations between parameters (Jelsch *et al.*, 2000 ▸) and helps in the deconvolution between thermal displacement and charge-density parameters.

Some properties may involve only part of the parameters, such as electrostatic interaction energy between molecular fragments. If this property depends only on a few atom parameters, the ‘square root matrix **S** calculation’ step [equation (11)] could in principle be performed considering the reduced normal matrix corresponding only to these specific atomic parameters. This will however lead to an underestimation of SSD values. It is therefore recommended that SSDs are obtained using a full normal matrix issued from all parameters. For this reason, thermal parameters should be taken into account in the normal matrix calculation when generating the perturbed structures, although they do not have a direct impact on the charge density.

## 5. Conclusion   

At the convergence of a least-squares crystallographic refinement against diffraction data, the e.s.d.’s of the parameters used to model the molecular structure and electron density can be directly retrieved. However, the uncertainties on derived molecular properties are not readily available. To estimate the errors of properties, series of models at ‘standard deviation’ from the final refined model can be easily generated by using vectors of random numbers and a square root of the inverted normal matrix. The SSDs obtained for the properties derived from a sample of such deviating structures can be used as estimated values of their uncertainties. For instance, samples of 20 perturbed structures yield SSD values with an expected relative precision of 16%. The average value of properties *P* in the perturbed models appears to be generally within one SSD from the final refined value; in the case of topological integrated charges and electrostatic energies, it was, for instance, found that 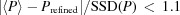.

In the BOH2 structure, the SSD of the electron density at the *X*—*Y* bond CPs is in the range 0.01 to 0.04 e Å^−3^, which represents 0.5 to 2% in relative value. The average SSD on the corresponding Laplacian values is 0.42 e Å^−3^ and the average relative error SSD(∇^2^ρ)/|∇^2^ρ| is 3%. The average uncertainty on the ellipticity ∊ on *X*—*Y* bond CPs is found to be around 0.01 and is usually not dependent on the ellipticity value ranging here from 0.002 to 0.33. For *X*—H bonds, the average SSD(∊) is 0.007, while the maximal value ∊ is 0.012. For interacting molecular dimers of the BOH2 molecule in the crystal, the error on the electrostatic energy is typically in the 2 to 4 kJ mol^−1^ range. Intermolecular topological bond paths were found to be stable and preserved in most of the 17 interactions, except for four weak contacts. The SSD of the electrostatic potential on the molecular surface lies between 5 × 10^−3^ and 2 × 10^−2^ e Å^−1^. High absolute values of electrostatic potential, which are usually interpreted as electronegative or electropositive sites, are shown to be significant with high signal-over-noise ratios.

The availability of estimated errors is important for the proper interpretation of experimental charge-density results, for instance, in the comparison of properties among similar chemical groups in a molecule, or of independent molecules in the asymmetric unit. Discrepancies found in the properties of chemically equivalent atoms or of covalent bonds are physically meaningful only if they are significantly larger than the estimated error.

The comparison of closely related but different compounds such as topological properties in different peptides as investigated by Flaig *et al.* (1999 ▸) and Grabowsky *et al.* (2008 ▸) is also more pertinent when an estimation of errors is available.

One should also recall that the actual errors obtained by the SSD method give information about the precision but may not take into account the effects of systematic errors on model accuracy. The structural and charge-density parameters may be driven away from their ‘true’ values to compensate for the systematic errors, while the crystallographic *R* factors may not be significantly worsened.

## Supplementary Material

Crystal structure: contains datablock(s) I. DOI: 10.1107/S2053273318004308/ae5043sup1.cif

Structure factors: contains datablock(s) I. DOI: 10.1107/S2053273318004308/ae5043Isup2.hkl

Supporting information file. DOI: 10.1107/S2053273318004308/ae5043Isup3.cml

Supporting figures and table. DOI: 10.1107/S2053273318004308/ae5043sup4.pdf

CCDC reference: 1829445

## Figures and Tables

**Table 1:** Crystal data and diffraction data collection statistics for the BOH2 molecule

Crystal data	
Chemical formula	C_11_H_15_BO_2_
Molecular weight	190.039
Crystal system, space group	Orthorhombic, *Pbca*
Temperature (K)	90 (1)
*a*, *b*, *c* (Å)	7.52004 ▸(9), 9.38374 (13), 30.7120 (5)
Volume (Å^3^), *Z*	2167.23 (5), 8
Radiation type	Mo *K*α
λ (Å)	0.71073
*F* (000)	816
Crystal shape and colour	Block and colourless
Crystal dimensions (mm)	0.34 × 0.18 × 0.10
Data collection	
Diffractometer	Rigaku MicroMax-007HF
Absorption correction	*CrysAlisPro 1.171.38.37f* †
Absorption coefficient μ (mm^−1^)	0.077
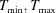	0.472, 0.999
sinθ_max_/λ (Å^−1^)	1.12
No. measured, unique reflections	117 942‡, 12 055
No. reflections (*I* > 2σ)	10 680
Completeness (%) at sinθ_max_/λ	96.5
*R*_int_	3.06%
Refinement	
Weighting scheme	*W*_*hkl*_ = 3.3/σ_*I*_^2^
*wR* ^2^(*I*), *R*(*F*)	3.62%, 2.70%
Goodness of fit	1.0

† Rigaku Oxford Diffraction (2015 ▸).

‡ At sinθ_max_/λ = 1.22 Å^−1^.

**Table 2:** Properties of critical points (CPs) for covalent bonds Bond lengths and distances of CPs to the two bonded atoms are given. The electron density, Laplacian value and ellipticity at CP positions are also reported. The SSD uncertainties are given in parentheses. Some remarkable values are indicated in bold. For the *X*—*Y* bonds (non-H atoms), the e.s.d.’s obtained by standard propagation error are also reported in brackets.

Atom		Distance (Å)						
*X*	*Y*	(*X*, *Y*)		(*X*, CP)	(*Y*, CP)	ρ(**r**_CP_) (e Å^−3^)	∇^2^ρ (e Å^−5^)	∊
C4	C5	1.3935 (3)	[4]	**0.68 (2)**	**0.72 (2)**	2.184 (8)	−20.7 (4)	0.21 (2)
C3	C4	1.3929 (3)	[4]	0.683 (9)	0.710 (9)	2.18 (2)	−20.3 (6)	0.222 (9)
C5	C6	1.3955 (3)	[3]	0.696 (9)	0.700 (9)	2.11 (2)	−19.5 (4)	0.25 (1)
C2	C3	1.3959 (4)	[4]	0.643 (8)	0.753 (8)	2.11 (2)	−19.1 (3)	0.20 (1)
C1	C6	1.3975 (2)	[3]	0.685 (8)	0.712 (7)	2.166 (9)	−19.8 (4)	0.235 (7)
C1	C2	1.4014 (3)	[3]	0.706 (8)	0.695 (8)	2.17 (2)	−19.3 (4)	0.20 (1)
C1	C7	1.5079 (2)	[3]	0.784 (6)	0.724 (6)	1.70 (2)	−11.5 (3)	0.019 (9)
C7	C8	1.5340 (2)	[3]	0.776 (5)	0.758 (5)	1.592 (7)	−9.9 (2)	0.101 (8)
C8	C9	1.5331 (2)	[3]	0.775 (5)	0.758 (5)	1.62 (1)	−9.9 (3)	0.15 (2)
C9	C10	1.5001 (2)	[2]	0.712 (6)	0.788 (6)	1.73 (1)	−13.2 (3)	0.17 (1)
C10	C11	1.3439 (2)	[2]	0.682 (8)	0.662 (8)	2.28 (2)	−21.6 (4)	0.334 (8)
B1	C11	1.5614 (2)	[2]	0.495 (3)	1.066 (3)	1.30 (2)	**−7 (1)**	0.09 (3)
B1	O1	1.3711 (2)	[2]	**0.4496 (6)**	**0.9217 (6)**	1.35 (1)	**19.8 (8)**	0.002 (7)
B1	O2	1.3762 (2)	[2]	**0.4519 (6)**	**0.9251 (6)**	1.33 (2)	**17.5 (9)**	0.03 (2)
C4	H4	1.066 (8)		0.701 (8)	0.366 (6)	1.78 (2)	−16.4 (5)	0.047 (6)
C5	H5	1.059 (7)		0.717 (7)	0.342 (6)	1.80 (2)	−17.7 (4)	0.065 (5)
C3	H3	1.060 (9)		0.726 (7)	0.334 (5)	1.80 (2)	−16.7 (4)	0.056 (6)
C6	H6	1.064 (7)		0.719 (9)	0.345 (5)	1.81 (2)	−17.2 (5)	0.056 (6)
C2	H2	1.063 (9)		0.726 (8)	0.337 (6)	1.80 (2)	−15.5 (3)	0.053 (6)
C7	H71	1.056 (6)		0.676 (6)	0.380 (5)	1.745 (8)	−13.5 (3)	0.031 (5)
C7	H72	1.063 (6)		0.687 (6)	0.376 (6)	1.74 (2)	−14.3 (4)	0.049 (4)
C8	H81	1.063 (7)		0.689 (5)	0.374 (6)	1.72 (2)	−14.6 (4)	0.048 (9)
C8	H82	1.046 (8)		0.679 (8)	0.367 (6)	1.758 (8)	−14.9 (4)	0.03 (2)
C9	H91	1.037 (8)		0.65 (1)	0.383 (8)	1.691 (9)	−14.0 (3)	0.09 (2)
C9	H92	1.050 (7)		0.674 (8)	0.376 (6)	1.65 (2)	−11.8 (4)	0.024 (8)
C10	H10	1.055 (7)		0.682 (7)	0.373 (5)	1.75 (1)	−16.8 (3)	0.084 (7)
C11	H11	1.045 (7)		0.679 (5)	0.366 (5)	1.748 (9)	−14.7 (4)	0.031 (9)
O1	H1	0.932 (9)		0.719 (5)	0.214 (6)	**2.36 (4)**	**−31 (1)**	0.006 (2)
O2	H2	0.936 (8)		0.724 (4)	0.211 (5)	**2.39 (3)**	**−31 (1)**	0.022 (2)

**Table 3:** Properties of critical points (CPs) of intermolecular interactions involving molecules at distance shorter than 3 Å from any atom of the reference BOH2 molecule Each CP is identified by its two major contributing atoms A1 and A2 which are linked by the corresponding bond path. Two CPs of weak interatomic contacts with ambiguous bond path are in bold (linked atoms are not stable). Some CPs are not detected with all deviating models (occurrence < 20) and are shown in italics. For each pair of major contributing atoms, their occurrence number, interatomic distance and distances between CP and atoms are given. The electron density, Laplacian ∇^2^ρ and ellipticity ∊ values at CP position are also reported. The SSD uncertainties are given in parentheses.

				Distance (Å)				
Symmetry code	A1	A2	Frequency	(A1, A2)	(A1, CP)	(A2, CP)	ρ_CP_ (e Å^−3^)	∇^2^ρ_CP_ (e Å^−5^)
(i)	O1	H2*O*	20	1.824 (7)	1.193 (4)	0.631 (9)	0.207 (5)	3.41 (8)
								
(ii)	O2	H1*O*	20	1.767 (9)	1.171 (4)	0.60 (1)	0.221 (7)	4.0 (2)
	H10	O1	20	2.580 (6)	1.086 (6)	1.521 (4)	0.055 (2)	0.801 (6)
								
(iii)	O1	H92	20	2.801 (5)	1.592 (3)	1.226 (4)	0.0345 (6)	0.489 (5)
								
(iv)	*H81*	*H91*	*13*	2.711 (9)	1.43 (2)	1.32 (2)	0.0185 (8)	0.256 (4)
	H81	C4	20	2.887 (4)	1.170 (5)	1.719 (7)	0.0504 (8)	0.580 (6)
	*H92*	*H11*	*17*	2.775 (9)	1.45 (2)	1.36 (2)	0.0109 (6)	0.176 (4)
	H71	H11	20	2.777 (7)	1.327 (9)	1.479 (5)	0.0153 (6)	0.227 (3)
	H71	H91	20	2.663 (6)	1.351 (7)	1.319 (8)	0.0230 (6)	0.307 (4)
								
(v)	C11	H5	20	2.939 (6)	1.768 (5)	1.217 (6)	0.0363 (7)	0.417 (3)
	O2	H6	20	2.906 (7)	1.737 (8)	1.210 (7)	0.026 (2)	0.368 (5)
								
(vi)	**C1**	**H4**	**13**	2.916 (9)	1.82 (1)	1.179 (7)	0.0364 (8)	0.452 (5)
	**C6**	**H4**	**7**	2.957 (6)	1.816 (4)			
	H71	H5	20	2.262 (8)	1.177 (8)	1.121 (7)	0.040 (2)	0.564 (7)
								
(vii)	H72	H3	20	2.376 (8)	1.194 (6)	1.182 (8)	0.035 (2)	0.483 (4)
	H2	H4	20	2.793 (8)	1.45 (2)	1.45 (2)	0.0146 (5)	0.198 (6)
								
(viii)	H2	C3	20	2.972 (7)	1.240 (7)	1.777 (7)	0.0353 (6)	0.408 (5)
	**H2**	**H4**	**17**	2.425 (5)	1.153 (6)	1.352 (7)	0.0432 (9)	0.528 (5)
	**H2**	**C3**	**3**	2.972 (7)				

Symmetry operators: (i) −*x*, −*y* + 2, −*z*; (ii) −*x* + ½, *y* − ½, *z*; (iii) −*x* + 1, −*y* + 2, −*z*; (iv) −*x* + 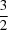, *y* − ½, *z*; (v) *x* − 1, *y*, *z*; (vi) −*x* + 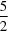, *y* − ½, *z*; (vii) −*x* + 2, *y* − ½, −*z* + ½; (viii) *x* − ½, *y*, −*z* + ½.

**Table 4:** Atomic charges in electrons along with their SSD values *Q*_topo_ (e) charges are integrated over the atomic basins of the molecule isolated from the crystal. *Q*_val_ = *N*_val_ − *P*_val_ are the atomic charges derived from the valence populations. The e.s.d. values are the estimated standard deviations of *P*_val_ directly derived from the full normal matrix inversion.

Atom	*Q*_topo_	SSD	*Q*_val_	e.s.d.	SSD
C1	−0.093	0.035	−0.017	0.054	0.067
C2	−0.048	0.038	−0.208	0.057	0.054
C3	−0.262	0.035	−0.128	0.063	0.036
C4	−0.116	0.048	−0.112	0.062	0.047
C5	−0.197	0.058	−0.227	0.059	0.059
C6	−0.160	0.045	−0.175	0.054	0.059
C7	0.020	0.034	−0.112	0.047	0.057
C8	−0.012	0.031	−0.25	0.048	0.051
C9	0.026	0.027	−0.241	0.048	0.051
C10	−0.024	0.028	−0.238	0.043	0.052
C11	−0.823	0.029	0.228	0.055	0.059
H2	0.094	0.015	0.129	0.024	0.019
H3	0.126	0.016	0.090	0.024	0.021
H4	0.068	0.017	0.064	0.025	0.024
H5	0.136	0.017	0.144	0.023	0.019
H6	0.110	0.025	0.131	0.022	0.027
H71	−0.005	0.010	0.069	0.023	0.021
H72	0.003	0.014	0.054	0.023	0.024
H81	0.060	0.014	0.159	0.019	0.020
H82	0.005	0.017	0.136	0.021	0.025
H91	−0.009	0.021	0.126	0.024	0.027
H92	0.043	0.013	0.163	0.021	0.026
H10	0.054	0.017	0.157	0.021	0.020
H11	0.067	0.013	0.022	0.021	0.023
H1O	0.584	0.012	0.355	0.015	0.014
H2O	0.561	0.008	0.325	0.015	0.010
O1	−1.326	0.019	−0.241	0.024	0.025
O2	−1.291	0.018	−0.237	0.024	0.020
B1	2.409	0.013	−0.171	0.057	0.048

**Table 5:** Total electrostatic interaction energy between interacting dimers in the crystal and the standard deviation in the sample The energy summation was performed with a unitary coefficient for all dimers except for the involutional symmetry operators (−*x*, −*y* + 2, −*z* and −*x* + 1, −*y* + 2, −*z*) which were counted as half. Non-involutional symmetry operators *f* form two equivalent dimers around the reference molecule, with operators *f* and *f* ^−1^. The SSDs were computed on 20 deviating models generated using the full least-squares normal matrix (‘SSD all parameters’) and using the reduced normal matrix obtained excluding the contributions of *U*_*ij*_ parameters (‘SSD no *U_ij_*’).

Symmetry	*E*_elec_ (kJ mol^−1^)	SSD all parameters	SSD no *U_ij_*
−*x*, −*y* + 2, −*z*	−62.2	4.2	5.1
−*x* + ½, *y* − ½, *z*	−37.2	3.2	2.7
−*x* + 1, −*y* + 2, −*z*	−16.5	1.7	1.7
−*x* + 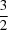 , *y* − ½, *z*	−9.1	3.0	2.0
*x* − 1, *y*, *z*	−1.1	2.0	1.7
−*x* + 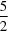 , *y* − ½, *z*	0.5	2.4	2.3
−*x* + 2, *y* − ½, −*z* + ½	2.4	1.8	1.2
*x* − ½, *y*, −*z* + ½	6.2	3.1	2.3
Sum	−77.7	14.8	8.9

## Appendix A

For any parameter *Y* distributed following a normal distribution with , if a sample of *N* events, , is taken from its distribution, the sample standard deviation *s*, biased estimator of , can be defined as

The quantity  follows a  probability distribution with  degrees of freedom, with a  sample variance. The standard deviation  of a χ probability distribution function with *k* degrees of freedom is defined as

Using this expression, the standard deviation  of the sample variance  distribution can be derived,

and, by propagation of the uncertainty, the standard deviation  of the sample standard deviation *s* distribution is approximated by

where  is the mean of the sample standard deviation *s* distribution.

As an estimator of , the expected value  of the sample standard deviation *s* is well known to be approximately equal to  and thus the relative standard deviation on  becomes

## Appendix B

The goal of this appendix is to discuss the way, after the convergence of an  parameter model refinement, to calculate the e.s.d.’s of only *m* parameters. The standard way is to derive the e.s.d.’s from the diagonal terms of the inverse matrix of the full  normal matrix **A**. To reduce computational resources, one could deduce e.s.d.’s starting from a principal  submatrix **U** of the normal matrix **A** corresponding to the *m* parameters considered. This second method will lead to underestimated e.s.d.’s as explained below. This demonstration uses the properties of symmetric positive-definite matrices.

In the simple case of a refinement with only two parameters (*x*_1_ and *x*_2_), the normal matrix can be written as:

where *r* is the ratio of the weighted scalar product between the two sets of intensity partial derivatives  and  [see equation (4)] and the product of their weighted norms. *r* can be considered as the cosine between two vectors and therefore −1 ≤ *r* ≤ 1.

The inverted normal matrix is then

One can note that −*r* is equal to the correlation coefficient between the two parameters. The e.s.d.’s are increased, as they are divided by () when the two parameters are refined together, illustrating the strong impact of a large parameter correlation.

In the general case, let us suppose the full normal matrix **A** is invertible, positive-definite and decompose it into

with **U** and **W** principal  and  submatrices of **A** corresponding, respectively, to the *m* parameters of interest and the *n* extra ones, and **V** its off-diagonal  submatrix.

As principal submatrices of the positive-definite matrix **A**, **U** and **W** are positive-definite matrices. The inverse matrix of **A**, noted , is also positive-definite and can be partitioned into four blocks as

where **C** and **E** are the positive-definite principal  and  submatrices, respectively, and **D** is the off-diagonal  submatrix.

The product  yields the identity matrix :

By identification, the following relations apply:

As mentioned previously, the submatrix **U** is invertible. Thus, the equations (27*b*)[Disp-formula fd27b] and (27*c*)[Disp-formula fd27c] imply, respectively, that the matrices **D** and **C** satisfy

Then, the combination of equations (28) and (29) yields

As a principal submatrix of the positive matrix **A** ^−1^, **E** is also a positive-definite matrix and its Cholesky decomposition is written, with **L** a lower triangular matrix, as follows:

Then, the combination of equations (30) and (31) gives

Let us define the  matrix **M** as ; equation (32) becomes

The diagonal elements of the product matrix , noted **T**, are positive numbers:

Therefore, the first *m* diagonal element of  is augmented compared with those of matrix :

Assuming GOF = 1, equation (35) implies the following inequality between the e.s.d.’s of the *m* parameters of interest derived from the full normal matrix **A**  and those derived from the reduced matrix **U** :

In other words, deducing the e.s.d.’s on the *m* parameters from a reduced normal matrix **U** leads to an underestimation of uncertainties. This conclusion is valid whatever the subset of parameters considered. One can note that in the particular case in which there were no correlations between the *m* parameters and the remaining *n* others, the matrix **V** would be equal to zero. As a result, **A** would be a block diagonal matrix, **M** = 0 and : the e.s.d.’s of parameters would not be underestimated if deduced from the reduced normal matrix **U**.
