# Supplementary material for: A method to estimate statistical errors of properties derived from charge-density modelling
Source: Acta Crystallogr A Found Adv. 2018 May 3;74(Pt 3):170–83. doi: 10.1107/S2053273318004308 (PMC5933404; doi:10.1107/S2053273318004308)
Supplement: Supplementary file 4 [file a-74-00170-sup4.pdf]

Acta Cryst A FOUNDATIONS ADVANCES

**Volume 74 (2018)**

**Supporting information for article:**

# A method to estimate statistical errors of properties derived from charge density modeling

**Bertrand Fournier, Benoît Guillot, Claude Lecomte, Eduardo C. Escudero-Adán and Christian Jelsch**

**Supplementary Materials.**

# Estimating errors of derived properties in charge density analyses

**Bertrand Fournier, Benoît Guillot, Claude Lecomte,**

**Eduardo C. Escudero-Adán & Christian Jelsch.**

**Acta Crystallographica A74 Advances (2018)**

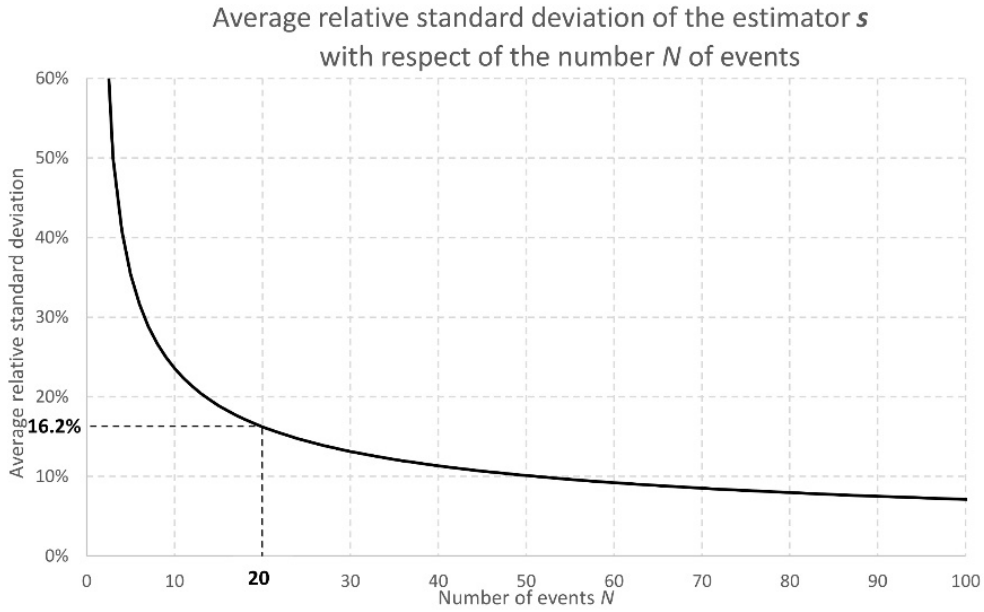


**Figure Sup1**. Plot of the relative standard deviation (equation (16)) of the SSD value as a function of the number $N$ of deviating models.

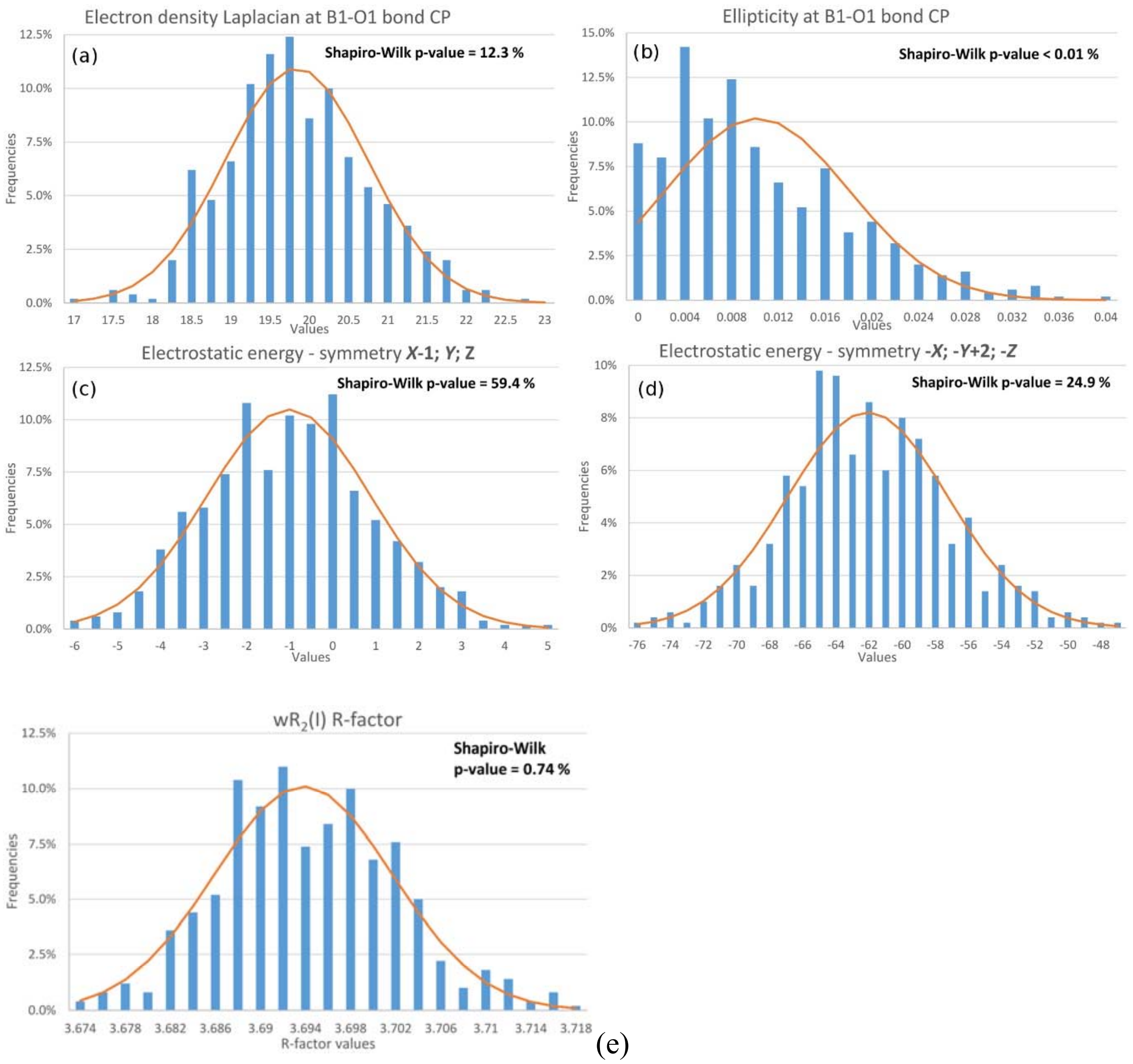

(e)

**Figure Sup2.**
Population histograms for different properties deduced from 500 perturbed models: electron density Laplacian (a) and ellipticity (b) at CP of B1-O1 bond, electrostatic interaction energies between reference and neighboring molecules (c) and (d). Ideal Gaussian distributions are defined considering sample average and SSD value, and superimposed. Shapiro-Wilk test *P*-value are also given.

(e) *R*-factor values $wR^2(I)$.

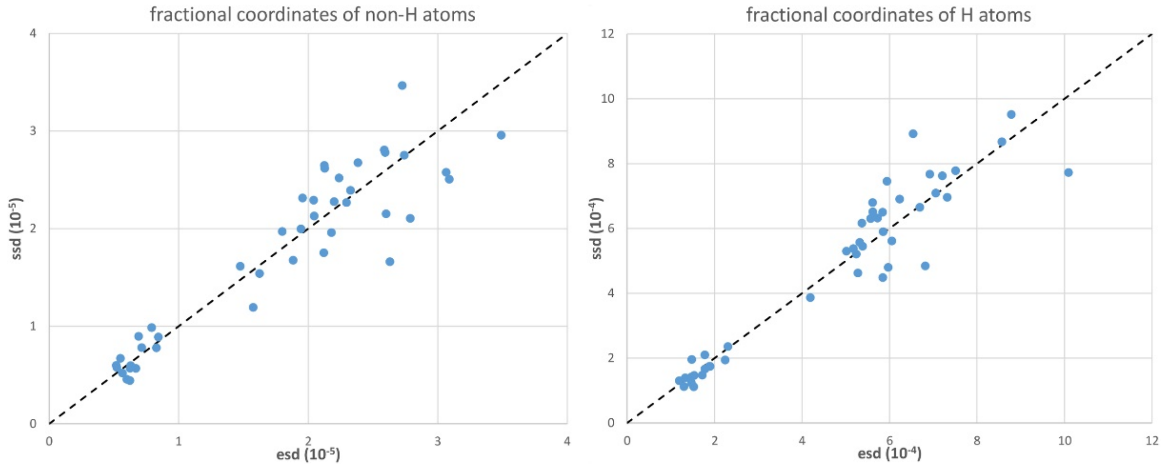


**Figure Sup3**. Scatterplot of e.s.d. (sigmaX) and SSD values for the fractional coordinates of the non-H atoms (left) and the H atoms (right) of the $C_{11}H_{15}BO_2$ molecule.

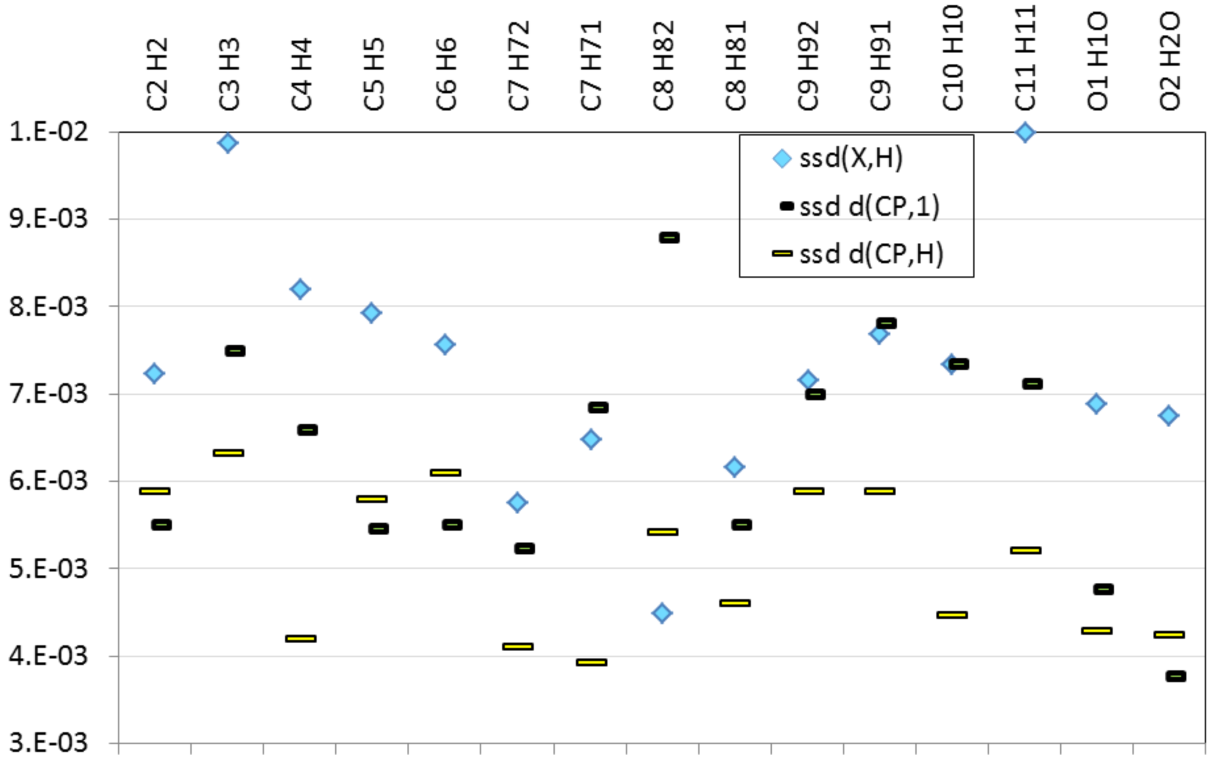


**Figure Sup4.** Plot of SSD values of X…CP and H…CP distances for all X-H covalent bonds. The SSD and e.s.d. (sigma) of the X-H bond distances are also shown.

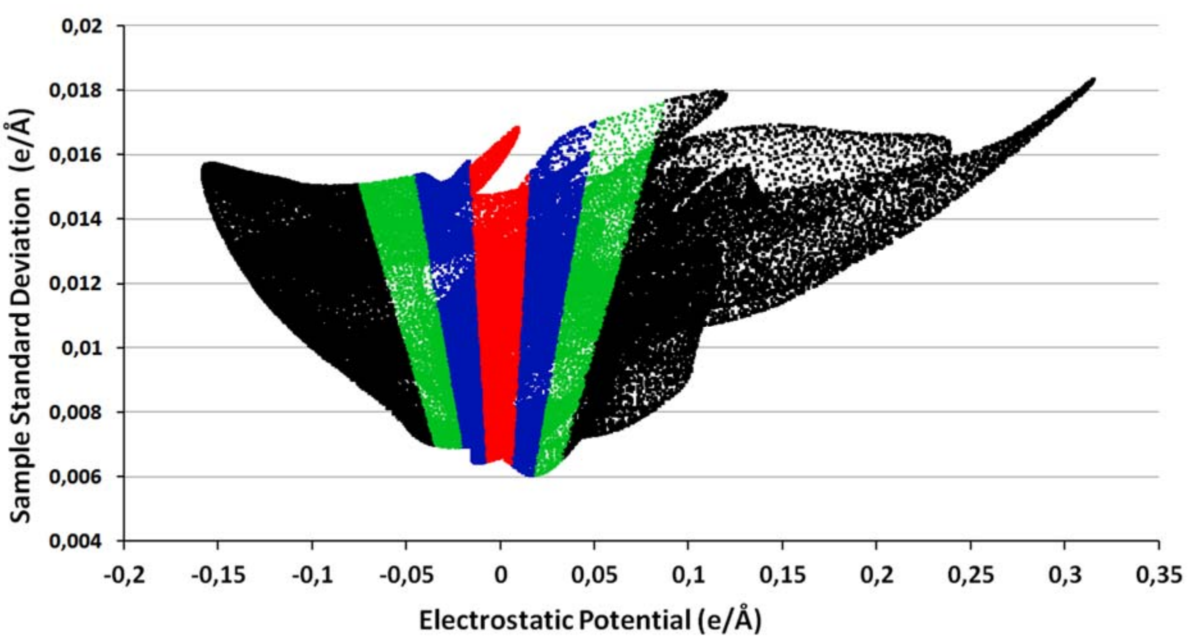


**Figure Sup5**. Sample standard deviation SSD($\varphi$) plotted versus the electrostatic potential values $\varphi$ on ~70k points defining the 0.001*au* electron density iso-surface of BOH2 compound. Dots are colored according to the signal over noise ratio $s$=|$\varphi$/SSD($\varphi$)|. Black : $s \geq 5$; green : $3 \leq s < 5$; blue : $1 \leq s < 3$ and red : $s < 1$.

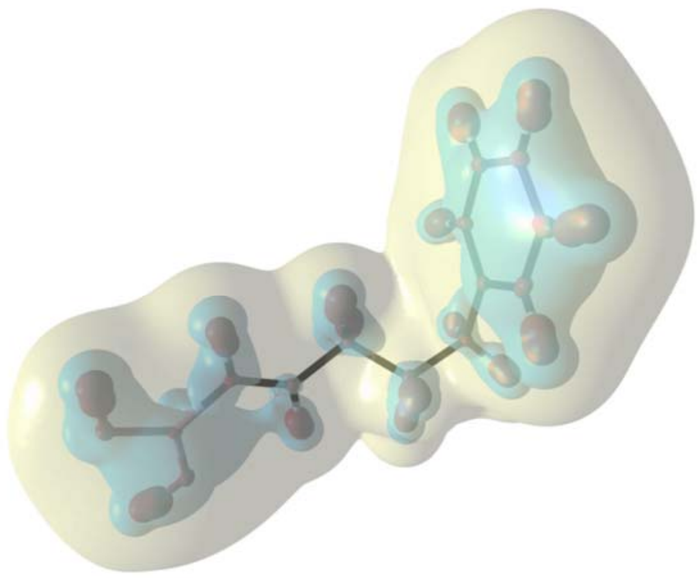

**Figure Sup6.** SSD($\varphi$) iso-contour surfaces of 0.04 (inner, red), 0.02 (intermediate, blue) and 0.01 e/Å (outer, yellow).

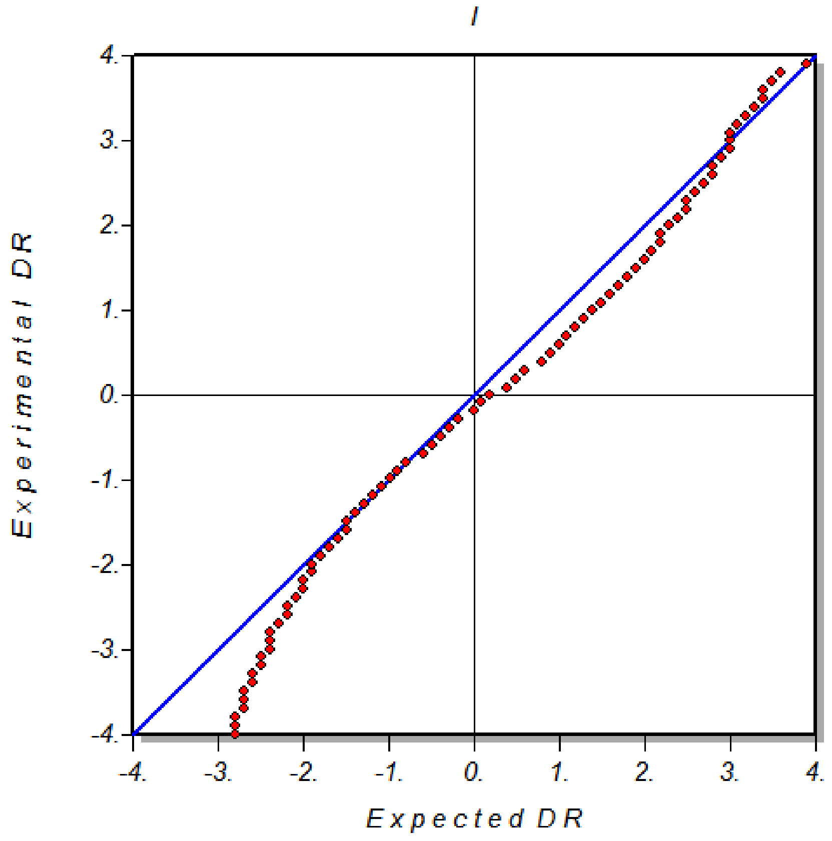


**Figure Sup7**. Normal probability plots of ($F^2{}_o$-$F^2{}_c$) using program XDRKplot ( Zhurov, Zhurova & Pinkerton, J. Appl. Cryst. (2008). 41, 340–349). The weighting scheme used is $\sigma^2 = [\sigma(F^2)]^2$.

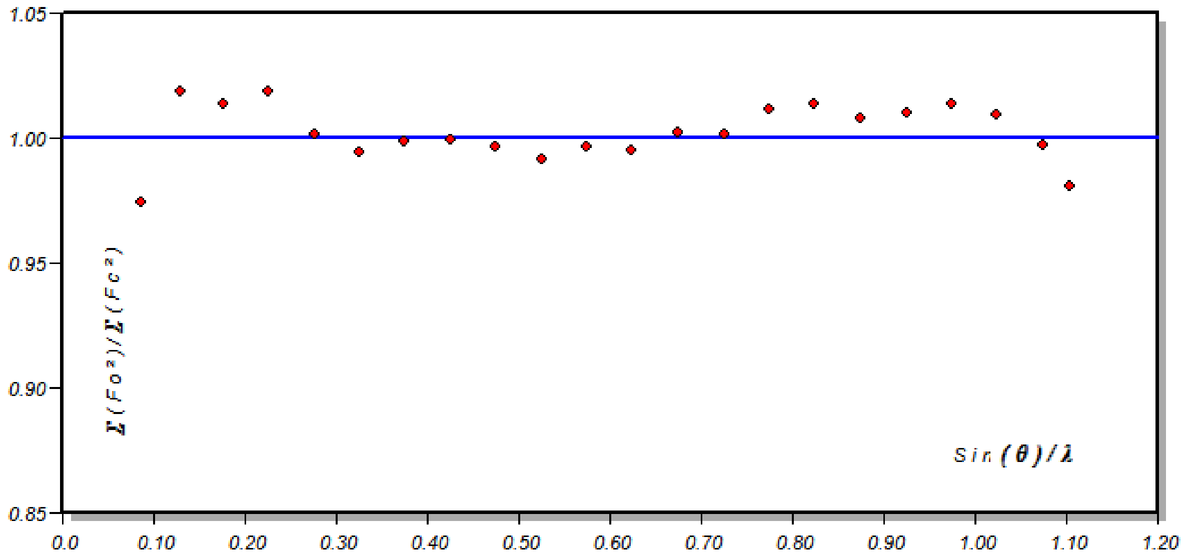


**Figure Sup8**. Variation of scale factor, $\Sigma (Fo^2)/ \Sigma (Fc^2)$, with respect to resolution for the BOH molecule.

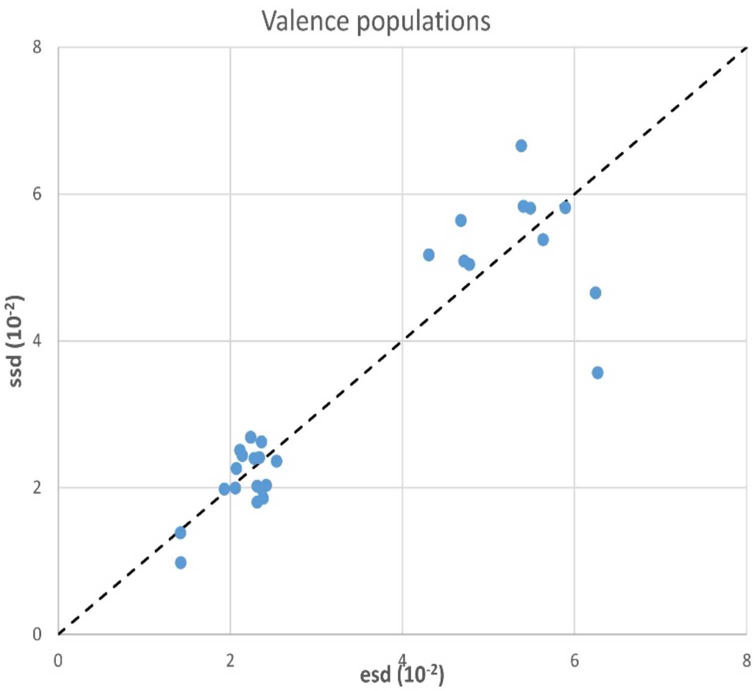


**Figure Sup9**.

Scatterplot e.s.d. vs. SSD values for the $P_{val}$ parameters of the $C_{11}H_{15}BO_2$ molecule model.

**Table Sup1.**

$\lambda_1$, $\lambda_2$, $\lambda_3$ eigenvalues of the Hessian matrix $\partial^2\rho/\partial x_i\partial x_j$ at the bond critical points of BOH2 molecule.

| Atom A | Atom B | $\lambda_1$ | SSD | $\lambda_2$ | SSD | $\lambda_3$ | SSD |
|---|---|---|---|---|---|---|---|
| B1 | C11 | -10.51 | 0.23 | -9.64 | 0.19 | 13.34 | 0.77 |
| B1 | O1 | -12.78 | 0.21 | -12.76 | 0.20 | 45.35 | 0.59 |
| B1 | O2 | -13.00 | 0.25 | -12.59 | 0.27 | 43.07 | 0.56 |
| C1 | C2 | -16.50 | 0.16 | -13.77 | 0.20 | 10.96 | 0.42 |
| C1 | C6 | -16.80 | 0.19 | -13.60 | 0.15 | 10.64 | 0.41 |
| C1 | C7 | -11.30 | 0.16 | -11.08 | 0.12 | 10.92 | 0.27 |
| C2 | C3 | -16.11 | 0.22 | -13.42 | 0.15 | 10.41 | 0.43 |
| C3 | C4 | -17.10 | 0.16 | -13.99 | 0.13 | 10.82 | 0.52 |
| C4 | C5 | -16.72 | 0.13 | -13.80 | 0.12 | 9.84 | 0.30 |
| C5 | C6 | -16.16 | 0.16 | -12.97 | 0.16 | 9.60 | 0.30 |
| C7 | C8 | -10.34 | 0.10 | -9.38 | 0.08 | 9.81 | 0.17 |
| C8 | C9 | -10.32 | 0.11 | -8.99 | 0.14 | 9.38 | 0.14 |
| C9 | C10 | -11.80 | 0.13 | -10.06 | 0.13 | 8.64 | 0.19 |
| C10 | C11 | -17.78 | 0.16 | -13.33 | 0.13 | 9.54 | 0.32 |
| C1 | H10 | -16.03 | 0.15 | -14.79 | 0.18 | 14.05 | 0.25 |
| C1 | H11 | -16.07 | 0.20 | -15.58 | 0.18 | 16.97 | 0.41 |
| C2 | H2 | -17.48 | 0.26 | -16.59 | 0.25 | 18.54 | 0.52 |
| C3 | H3 | -17.95 | 0.27 | -17.00 | 0.28 | 18.24 | 0.59 |
| C4 | H4 | -16.40 | 0.24 | -15.66 | 0.25 | 15.62 | 0.43 |
| C5 | H5 | -17.46 | 0.26 | -16.40 | 0.28 | 16.16 | 0.43 |
| C6 | H6 | -17.59 | 0.20 | -16.65 | 0.22 | 17.05 | 0.55 |
| C7 | H71 | -14.97 | 0.22 | -14.52 | 0.22 | 15.97 | 0.47 |
| C7 | H72 | -15.19 | 0.21 | -14.48 | 0.19 | 15.39 | 0.36 |
| C8 | H81 | -15.19 | 0.26 | -14.50 | 0.27 | 15.07 | 0.43 |
| C8 | H82 | -15.49 | 0.19 | -15.04 | 0.27 | 15.62 | 0.55 |
| C9 | H91 | -14.22 | 0.23 | -12.99 | 0.24 | 13.18 | 0.48 |
| C9 | H92 | -13.47 | 0.17 | -13.16 | 0.14 | 14.80 | 0.40 |
| H1 | O1 | -38.14 | 0.99 | -37.91 | 0.98 | 44.90 | 1.55 |
| H2 | O2 | -38.82 | 0.86 | -37.97 | 0.85 | 45.74 | 1.33 |
